# Disentangling eye movement desensitization and reprocessing mechanisms of action: The impact of eye movements in the eye blink conditioning task

**DOI:** 10.1111/papt.70000

**Published:** 2025-07-03

**Authors:** Daniel Folch‐Sanchez, Chrysanthi Blithikioti, Laura Nuño, Pablo Barrio, Roger Borràs, Laura Blanco, Flavia Piazza, Mercè Balcells‐Oliveró, Laia Miquel

**Affiliations:** ^1^ Health and Addictions Research Group; Addictions Unit. Psychiatry and Psychology Service, ICN Hospital Clinic Barcelona Barcelona Spain; ^2^ Institut d'Investigacions Biomèdiques August Pi i Sunyer (IDIBAPS) Barcelona Spain; ^3^ University of Barcelona Barcelona Spain; ^4^ Red de Investigación en Atención Primaria en Adicciones (RIAPAd) Spain; ^5^ Department of General Psychology University of Padova Padova Italy

**Keywords:** conditioned response, eye blink conditioning task, eye movement desensitization and reprocessing, eye movements, fear extinction learning

## Abstract

**Objectives:**

Eye movement desensitization and reprocessing (EMDR) is an effective evidence‐based treatment for post‐traumatic stress disorder. However, the therapeutic mechanisms underlying eye movements (EM) remain unclear. This study aimed to investigate the effect of horizontal EM on fear extinction learning in healthy individuals, using an Eye Blink Conditioning (EBC) task. This experimental paradigm has been widely used to explore associative fear learning and memory as a form of classical conditioning.

**Methods:**

Healthy participants were included to the study protocol and divided randomly into two groups. The EM group (*n* = 20) were asked to follow horizontally the experimenter's moving finger at the beginning of the extinction phase and the control group (*n* = 19) did not engage in any specific task. Sociodemographic and clinical information was collected. Percentage of conditioned response (CR) occurrence, time of onset and intensity between and within groups over longitudinal time were analysed using generalized multilevel mixed effects for repeated measures.

**Results:**

Results showed accelerated extinction learning in the EM group, with an 18.2% probability of CR occurrence in the first block of extinction, compared to the control group (40.9%) (*p*‐value = .007).

**Conclusions:**

The findings indicate that horizontal EM accelerates the extinction process in the EBC task. Therefore, this paradigm, used for studying associative learning and memory, could serve as an objective measure to investigate the mechanisms of action involved in desensitizing traumatic experiences during EMDR treatment.

## INTRODUCTION

Eye movement desensitization and reprocessing (EMDR) is an effective evidence‐based treatment for post‐traumatic stress disorder (PTSD) (Shapiro, 1989), recommended by the American Psychiatric Association (APA) (Benedek et al., 2009) and the World Health Organization (WHO) (World Health Organization, 2013). In this therapy, patients recover their traumatic memories while concurrently directing their attention towards an external stimulus (Calancie et al., 2018; Rousseau et al., 2020). Specifically, this therapeutic technique involves the utilization of bilateral sensory stimulation (BLS), with lateral eye movements (EM) being the most common form used during revisiting past distressing events. As a result, individuals frequently report a marked reduction in the intensity of both the vividness and emotional charge associated with their traumatic experiences (Calancie et al., 2018), achieved through the desensitization of the cognitive, emotional and physical aspects of distress (Chen et al., 2014; Rousseau et al., 2020). Despite the acknowledged effectiveness in mitigating symptoms of PTSD, the exact mechanisms of action of EMDR still remain unclear.

Emerging evidence indicates that EM plays a significant role in the efficacy of memory desensitization within EMDR treatment (Christman et al., 2003; De Jongh et al., 2013; Engelhard et al., 2010; Engelhard et al., 2011; Gunter & Bodner, 2008; Schubert et al., 2011; Wadji et al., 2022). In these studies, participants were told to recall negative memories that elicited feelings of fear or distress, followed by exposure to EM or a control condition. Subsequently, subjects rated the emotionality, vividness, distress and recall difficulty associated with the traumatic memory. The findings consistently demonstrated that EM can effectively reduce both the perceived vividness and emotional intensity during the recall of traumatic stimuli and negative memories. However, these studies relied on subjective ratings provided by participants, which may introduce methodological limitations due to the absence of objective measurements.

In contrast, only a few investigations have employed objective measurements to comprehend the mechanisms of action underlying EM in humans. These studies employed a fear conditioning and extinction paradigm, a widely used approach to uncover the underlying mechanisms of fear‐related deficits in PTSD (Boukezzi et al., 2017). Incorporating BLS, such as EM, during fear extinction learning has been proposed as a valuable model to explore the effects of EM within the context of EMDR therapy (Boukezzi et al., 2017). For instance, Szeska et al. (2023) employed a fear conditioning and extinction protocol involving electric shocks. The findings of this study indicated that saccadic EM, in contrast to smooth EM, led to a reduced defensive response activation, measured through fear‐potentiated startle responses and fear bradycardia. Similarly, Boukezzi et al. (2017) demonstrated the effectiveness of bilateral auditory stimulation in reducing skin conductance responses within a classical fear conditioning and extinction paradigm. These findings also correlated with a decrease in fear expectations. Overall, while there is increasing evidence suggesting that EM contribute to memory desensitization, further studies utilizing objective measurements are needed to elucidate the underlying mechanisms of action of EM in EMDR treatment.

The classical eye blink conditioning (EBC) task is an objective and standardized task that could be used to prove the mechanisms of action underlying EM. The EBC task, rooted in Pavlovian conditioning principles, is based on the closure of the eyelid triggered by a corneal air puff. This air puff, denoted as the unconditioned stimulus (US), is consistently paired with a neutral stimulus (NS), represented by a tone. Over time, the tone transitions from a neutral stimulus to a conditioned stimulus (CS), inciting a conditioned learning response (CR) akin to the response evoked by the US. After this conditioning phase, participants enter an extinction phase wherein the US is no longer presented. The task aims to assess the number of trials it takes for participants to suppress the previously established associative learning between the US and the CS (Allen et al., 2019; Lonsdorf et al., 2017; Robleto et al., 2004). The feasibility, ethical considerations and well tolerance of the EBC task have been well established (Konrad et al., 2020). It has proven effective in exploring fear conditioning in patients with schizophrenia (Forsyth et al., 2012; Lundin et al., 2021; Sears et al., 2000), healthy individuals (Inoue et al., 2020) and those with PTSD (Allen et al., 2019). Notably, patients with PTSD have exhibited accelerated conditioning and delayed extinction learning during the EBC task (Allen et al., 2019). This makes the EBC task a suitable and reliable tool for assessing the contribution of EM in reducing the distress associated to aversive stimuli.

In this study, we employed the extinction learning paradigm of the EBC task to objectively measure the impact of EM. The primary objective was to investigate whether a straightforward and standardized EM intervention could enhance extinction learning in healthy subjects. We hypothesized that the process of extinction would be accelerated when healthy individuals engage in EM during the extinction phase (Boukezzi et al., 2017; Szeska et al., 2023), as opposed to control participants that performed no task. Demonstrating that EM can facilitate fear extinction learning could hold significant implications for understanding the underlying process and advancing our comprehension of the efficacy of EMDR therapy as a treatment for PTSD.

## METHODS

### Study design

The current study is a single‐blinded controlled trial designed to investigate the impact of EM in the context of extinction learning within the EBC task. Recruitment was performed between April 2022 and February 2023. This study received approval from the Ethics Committee of Hospital Clínic de Barcelona under the reference code HCB/2021/0283.

### Study population

A total of 39 healthy subjects were recruited for this study using the snowball sampling method. Inclusion criteria encompassed (1) individuals between the ages of 18 and 65 years (2) who signed the informed consent. Exclusion criteria were defined as (1) individuals with prior exposure to EMDR treatment or EM experiments, (2) individuals with a positive PTSD diagnosis or other Axis I disorders, as determined by the MINI International Neuropsychiatric Interview (Sheehan et al., 1998), as well as (3) those with hearing or visual impairments.

### Experimental protocol

Participants were recruited to take part in the study. First, the subjects signed the informed consent, wherein the confidentiality of their data is assured. Second, a psychologist collected sociodemographic information, including gender and age, along with clinical data related to their previous traumatic experiences. These variables were measured by using the Childhood Trauma Questionnaire (CTQ) (Bernstein & Fink, 1998) and the Life Events Checklist for DSM‐5 (LEC‐5) (Weathers et al., 2013). The CTQ provides scores for emotional abuse, physical abuse, sexual abuse, emotional neglect and physical neglect experienced during childhood and adolescence. Scores above 8, 7, 5, 9 and 7, respectively, on each subscale suggest low severity for each type of trauma. The LEC‐5 questionnaire investigates the presence or absence of various types of traumatic situations throughout an individual's lifetime. Also, participants underwent assessment for PTSD diagnosis and any other psychiatric comorbidities with the MINI.

Those individuals who met the criteria requirements proceeded to engage in the EBC task on the same day. The task was divided into two distinct phases: the acquisition phase (7 blocks), and the acquisition reinforcement (1 block) directly followed by the extinction phase (3 blocks). The EBC session lasted for about 30 min in total, with a 2–5‐min intermission between the two phases for both groups.

Participants were randomly assigned to one of two conditions during the extinction phase, using a sequential 1:1 randomization process. It is important to note that participants remained unaware of their treatment allocation. Specifically, participants in the experimental group (*n* = 20) underwent EM by following the researcher's fingers during the initial 30 s of the extinction phase, with all participants subjected to the same speed and duration. In contrast, participants in the control group (*n* = 19) were instructed to gaze directly at a specific point on the white wall situated in front of them.

**FIGURE 1:**
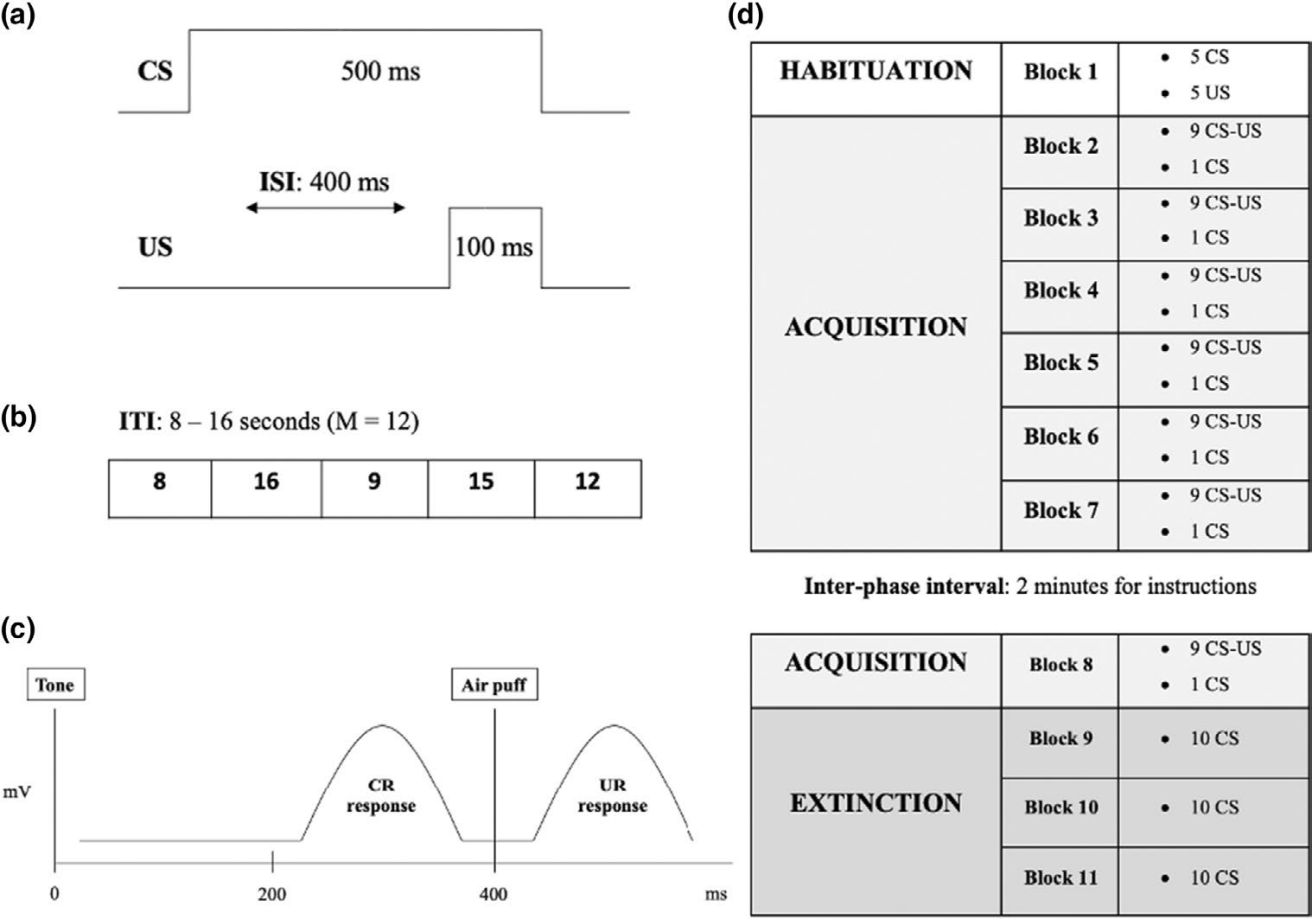
(a) CS‐US paired trial. (b) Intertrial interval (ITI). (c) Example of trial showing the conditioned (CR) and unconditioned (UR) responses. (d) Eye blink session protocol.

The EBC task was performed with a portable human eye blink conditioning system designed by San Diego Instruments. The system includes an infrared reflective sensor for recording eye blinks, glued together with small 1,5 mm air‐delivering tubing. The EBC device also comprises a headset sound‐delivering unit. Both the portable air puff and the headset sound‐delivering unit control the timing and the intensity of the US and CS, respectively. The EBC device was positioned so that the portable air puff was exactly located beneath the superior eyelid of the subject. The sound, air puff and position of the infrared reflective sensor were tested before the start of the session. A continuous background noise of 600 mV, which corresponds to approximately 46 dB, was delivered between trials to avoid participants from getting distracted during the experiment. The US consisted of a 100 ms air puff of 7 psi, which was enough to produce eyelid closure in 100% of the trials with no pain. The CS comprised a 500 ms pure tone of 900 mV (82.5 dB approximately), which was significantly higher in comparison with the background noise delivered during the session. Delay conditioning was used for the CS‐US paired trials (Lonsdorf et al., 2017; Skosnik et al., 2008): The 500 ms CS co‐terminated with the 100 ms US, producing a 400 ms interstimulus interval (ISI) (Figure 1a). Intertrial interval (ISI) ranged from 8 to 16 s (*M* = 12) (Figure 1b). Eye blinks occurring within the 200–400 ms time frame were coded as CR, while eye blink responses after 400 ms were coded as unconditioned responses (URs), elicited directly by the air puff (Parker et al., 2012) (Figure 1c). During the conditioning phase of the experimental protocol, subjects viewed a silent movie with the aim to maintain similar levels of arousal among participants. Overall, participants engaged in a 110‐trial EBC paradigm, divided into 11 blocks, which is a similar length compared to previous studies with related purposes (Steiner et al., 2019). Initially, participants were exposed to 5 CS and 5 US alone trials for habituation. The acquisition phase consisted of six blocks of 10 trials each. Each block contained nine paired CS‐US trials and one CS trial interspersed. Finally, after the inter‐phase interval, subjects received: First, one more block of acquisition, with the aim to reinforce the conditioning, and second, 30 extinction trials consisting of 30 CS trials while performing EM or no task, depending on their condition group (Figure 1d).

### Study outcomes

The primary outcome was the extinction learning differences between groups. A lower count of CR during the initial block of the extinction phase indicates a more rapid extinction learning process. CR differences between groups during the conditioning phase were also measured to control for potential bias. Secondary outcomes include specific properties of the eye blink CR, such as the time of onset of CR and the intensity of eye closure. A longer latency CR, meaning a delayed occurrence of the CR after the onset of the CS, is considered adaptive because its peak amplitude aligns with the onset of the US (Christian & Thompson, 2003). In terms of amplitude, higher peaks are observed when the CS‐US pairing occurs (Christian & Thompson, 2003). Therefore, the time of onset and intensity of the CR means per block were also analysed. Also, the potential interaction between previous traumatic experiences and the performance in the EBC task and between groups was also considered. Finally, UR differences in occurrence and intensity analysis were also included.

### Sample size and behavioural analysis

Our sample size was determined based on the methodology used in Inoue et al. (2020), which obtained significant effects using a similar paradigm with 20 participants per group in a five‐group design. In addition, with a significance level of *α* = 0.05, targeting a medium effect size (*f* = 0.25), assuming a correlation among repeated measures (*r* = .35), and aiming for a statistical power of 0.9, we calculated the same number of participants. Applying these criteria to our two‐group design, a total of 40 participants was required.

Sociodemographic and clinical continuous variables were presented as the mean value and standard deviation, and means between groups were compared using either the Student's t‐test or the Wilcoxon rank‐sum test as deemed appropriate. Sociodemographic and clinical categorical variables were expressed as total numbers with percentages and compared between groups utilizing Pearson's chi‐square test or Fisher's exact test as appropriate. CR occurrence, time of onset and intensity between and within groups over longitudinal time were analysed using generalized multilevel mixed effects for repeated measures. Odds ratios were computed to compare the extinction learning differences between groups per block, and the percentage of CR occurrence across the blocks was also reported. In all models, participant identification was treated as a random effect to account for within‐participant correlations. Model fitting utilized the Lme4 R package (v. 1.1.31), and model validation was performed using residual plots. Statistical comparisons were conducted with the emmeans library (v. 1.8.2). All analyses considered a two‐tailed type 1 error of 5%. The entirety of the analyses was executed using R (v. 4.2.2). PTSD is known to influence performance in the EBC task (Allen et al., 2019). Therefore, we checked for potential correlations between the percentage of CR occurrence and the clinical data on previous traumatic experiences and also tested the interaction between these variables, the group condition and the percentage of CR occurrence. Still, participants diagnosed with PTSD were ineligible for the study. The same correlation and interaction analyses were performed for sex and age.

## RESULTS

The analysis involved a total of 34 individuals (21 female). Data from three participants in the experimental group and two in the control group were excluded due to incorrect recording in the EBC software, leaving 17 participants in each group. The average age of participants was 33.1 years (*SD* = 10.4). No significant differences were observed between the control and the experimental group in terms of age, sex and the subscales of the CTQ questionnaire (including emotional, physical and sexual abuse, as well as emotional and physical neglect). Mean values for all CTQ subscales were below the cut‐off for abuse levels in both groups, with no significant differences observed. The number of total traumatic events, as provided by the LEC‐5 questionnaire, also showed no significant variations between the groups (Table 1). As per the exclusion criteria, none of the participants had a PTSD diagnosis or any other psychiatric condition.

**TABLE 1:** This table provides descriptive statistics and analysis of variance for age, along with the total scores of each CTQ subscale and the total number of traumatic events experienced obtained from the LEC‐5 questionnaire. Additionally, chi‐square differences for sex between groups are presented.

	EM Group (*n* = 17)	Control group (*n* = 17)	*F*‐statistic/chi‐square	*p*‐value (sig.)
	Mean (*SD*)	Mean (*SD*)		
Age	30.0 (7.7)	36.2 (12.2)	3.2	.084
Sex	**Female**: 12 (70.6%)	**Female**: 9 (52.9%)	1.1	.290
	**Male**: 5 (29.4%)	**Male**: 8 (47.1%)		
CTQ				
Emotional abuse	6.8 (3.2)	6.2 (1.5)	0.6	.459
Physical abuse	5.4 (1.0)	5.5 (0.9)	0.1	.718
Sexual abuse	5.1 (0.2)	5.0 (0.0)	1.0	.325
Emotional neglect	8.1 (3.5)	7.2 (2.4)	0.7	.399
Physical neglect	5.9 (1.3)	6.1 (1.7)	0.2	.654
LEC‐5: Total number of traumatic events	0.9 (1.1)	1.1 (1.0)	0.219	.643

### Differences in extinction learning between groups in the EBC task

In the extinction phase, significant differences were observed in the first block of extinction (block 9) between groups (OR: 0.32; 95% CI: 0.14, 0.73; *p* = .007) (Table 2). Participants who underwent EM during the extinction phase exhibited a lower occurrence of CR (18.2%) compared to the control group (40.9%). This indicates that participants in the EM group showed a more rapid extinction learning than the control group.

**TABLE 2:** Differences in CR likelihood across blocks and groups.

Block	Odds ratio	SE	IC (asymp.LCL, asymp.UCL)	*p*‐value
1	3.00	2.30	(0.67, 13.46)	.151
2	0.68	0.28	(0.30, 1.51)	.339
3	0.33	0.14	(0.15, 0.74)	.007***
4	0.76	0.31	(0.34, 1.70)	.503
5	0.54	0.22	(0.24, 1.21)	.137
6	0.72	0.30	(0.32, 1.61)	.427
7	0.59	0.24	(0.26)	.196
8	0.79	0.32	(0.35, 1.76)	.564
9	0.32	0.13	(0.14, 0.73)	.007***
10	0.47	0.20	(0.20, 1.09)	.080
11	0.73	0.31	(0.32, 1.67)	.455

**FIGURE 2:**
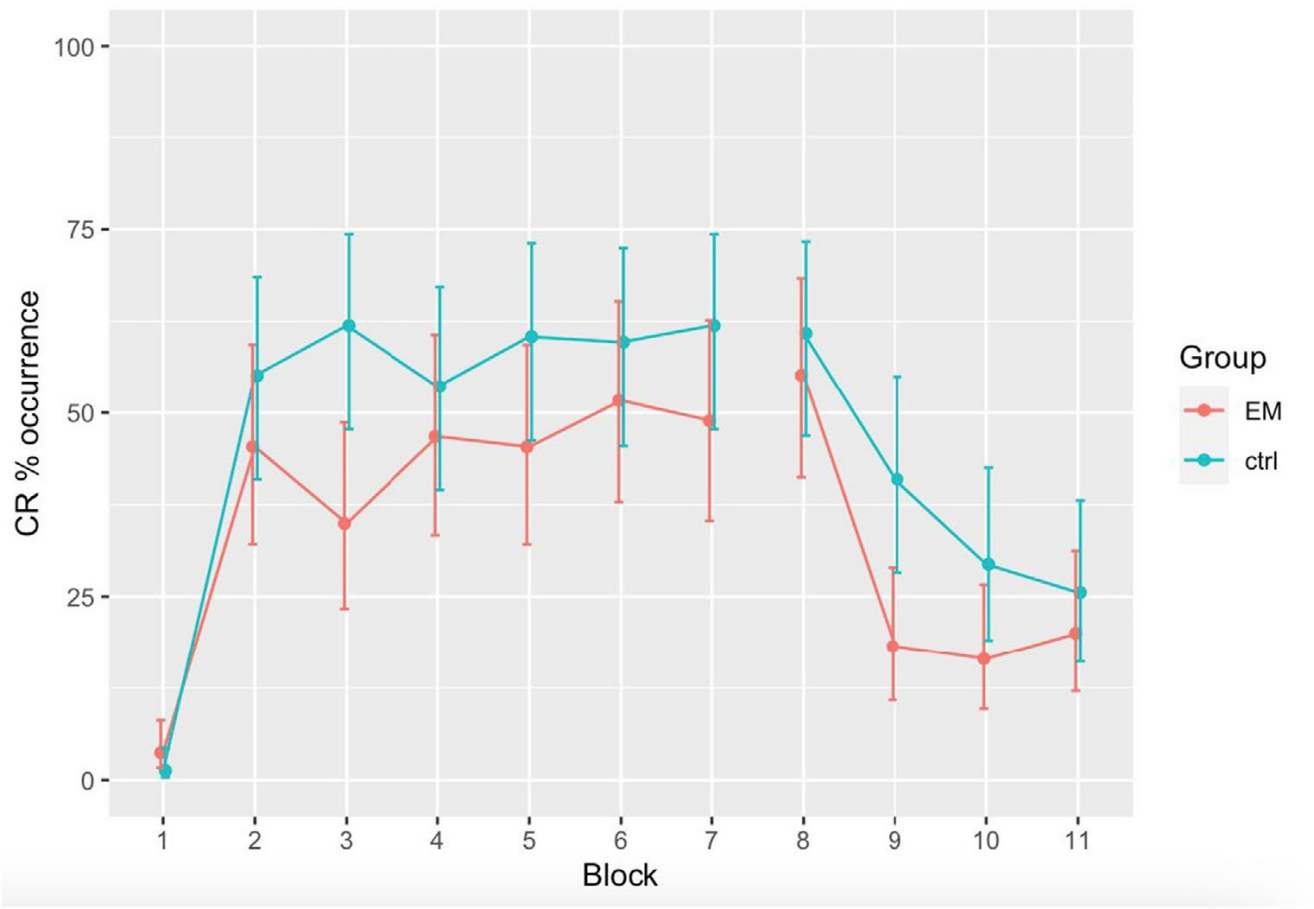
CR% occurrence across blocks for both the EM and control groups.

Regarding the conditioning phase, no substantial differences were observed among blocks, except for block 3 (EM group: 34.9%; control group: 61.9% of CR occurrence). CR percentages of occurrence for both groups are depicted in Figure 2.

### Comparison of time of onset and intensity (or amplitude) of CR between groups in the EBC task

The comparison of CR occurrence times between groups throughout the extinction phase revealed no statistically significant differences between groups across the analysed blocks (block 9, *p* = .173; block 10, *p* = .725; block 11, *p* = .829). Likewise, the examination of variations in the intensity of eyelid closure during the Eye Blink Conditioning (EBC) task did not yield any statistically significant findings between groups across the three blocks in the extinction phase (block 9, *p* = .431; block 10, *p* = .427; block 11, *p* = .932). Additionally, no significant differences between groups were observed in the time of onset and intensity during the conditioning phase.

### Exploring predictors: Correlation of age, sex and previous traumatic experiences with probability of CR

Age, sex and previous traumatic experiences (emotional abuse, physical abuse, sexual abuse, emotional neglect and physical neglect) were individually examined for their potential influence on CR occurrence during the extinction phase. However, none of these variables showed a statistically significant effect on CR: age (*p* = .8068), sex (*p* = .9383), emotional abuse (*p* = .449), physical abuse (*p* = .940), sexual abuse (*p* = .959), emotional neglect (*p* = .355) and physical neglect (*p* = .699). The estimated coefficients and standard errors for these variables further supported the absence of a meaningful impact on the probability of CR.

### Outcome 4: Analysing interactions: Impact of age, sex and previous traumatic experiences on probability of CR and treatment allocation

This investigation also aimed to assess potential associations between age, sex and previous traumatic experiences (emotional abuse, physical abuse, sexual abuse, emotional neglect and physical neglect) and their interaction with the treatment group concerning the probability of CR, to measure whether these variables influence the effectiveness of the intervention. None of the variables showed statistically significant effects on both group allocation and probability of CR (*p* > .05). Consequently, it can be inferred that age, sex, emotional abuse, physical abuse, sexual abuse, emotional neglect and physical neglect did not have a significant relationship with the group in predicting CR occurrence.

### Differences in the probability of UR and UR intensity between groups during the conditioning phase in the EBC task

No significant association was found between group allocation and the probability of UR during the conditioning phase (*F*(1, 2318) = 0.5766, *p* > .05). Additionally, no significant differences were observed in the intensity of UR between groups (*F*(1, 2318) = 0.6398, *p* > .05).

## DISCUSSION

The current study aimed to investigate whether a straightforward and standardized EM intervention, in comparison with a no‐task condition, could accelerate extinction learning during the EBC task in healthy individuals. As the EBC task has been established to be a reliable tool for evaluating fear learning in different mental conditions (Forsyth et al., 2012; Lundin et al., 2021; Sears et al., 2000), our investigation aimed to assess the potential impact of EM on fear learning. This study represents a step forward in elucidating the mechanisms underlying EMDR treatment.

Our findings suggest that EM intervention enhances extinction learning, evidenced by significant differences in the first block of extinction learning between the EM group and the control group (outcome 1). The control group exhibited a higher likelihood of CR (40.9%) compared to the EM group (18.2%) within block 9, indicating an accelerated extinction learning with a simple EM intervention. These results align with previous studies demonstrating the effectiveness of EM in reducing fear associated with aversive stimuli (Castelnuovo et al., 2019; de Voogd et al., 2018; Schubert et al., 2011). Furthermore, according to the criterion established by Konrad et al. (2020), conditioning can be assumed when participants have a CR occurrence of 50% per block. Both groups exhibited a CR percentage above 50% in the block preceding the extinction phase, indicating that conditioning had occurred. Applying this criterion, we infer that the control group also reached extinction in the first block of this phase (40.9%). However, the substantial reduction of CR occurrence in the EM group to 18.2% suggests that extinction occurred more rapidly, indicating that a simple EM intervention is highly effective in accelerating extinction learning in the EBC task among healthy individuals.

Importantly, no differences were observed in CR onset and CR intensity between the two groups in the extinction phase. The consistent timing of CR onset across both groups suggests that all participants exhibited a comparable response to the task. This uniformity could be attributed to the absence of any participants with a PTSD diagnosis, a factor known to potentially elicit maladaptive responses (Allen et al., 2019; Christian & Thompson, 2003). Therefore, we did not anticipate changes in the time of onset of the CR. Secondly, the fact that EM accelerated the extinction in CR occurrence in the first block of extinction did not translate in changes in the intensity of eyelid closure. The expected reduction in CR intensity as the extinction learning process unfolds (Christian & Thompson, 2003; Sears et al., 2000) was observed. However, this reduction did not result in differences between groups. Considering that both groups met the extinction criterion of CR occurrence below 50% (Konrad et al., 2020), the absence of differences in CR intensity between groups may be attributed to the fact that both groups achieved rapid extinction learning in the initial block of the extinction phase.

Similarly, no significant associations were found between group allocation and the probability and intensity of UR during the conditioning phase. This suggests that the observed differences in CR likelihood between groups during the extinction phase are not influenced by variations in UR during the initial stages of the conditioning phase.

Exploring potential predictors, including age, sex and previous traumatic experiences, revealed no significant effects on CR likelihood. This implies that, within the scope of this study, these variables did not play a substantial role in influencing the probability of CR during the extinction phase. First, no significant associations with age were anticipated, as prior studies have demonstrated successful acquisition and extinction rates across all age groups (Konrad et al., 2020). Second, no significant effects on sex were expected, in line with studies on rodents that have shown no notable differences in extinction learning based on sex (Thanellou et al., 2009; Thomas & Tran, 2012). Third, we found no correlation between previous traumatic experiences and CR occurrence. Controlling for the effect of previous traumatic experiences was important for the aim of this project, as PTSD is known to impact EBC performance (Allen et al., 2019). Additionally, the analysis of interactions aimed to understand the impact of age, sex and previous traumatic experiences on CR probability in conjunction with treatment condition. However, none of these variables demonstrated significant effects, reinforcing the notion that EM's influence on CR is not substantially mediated by these factors in healthy individuals.

## LIMITATIONS

Two important limitations should be considered when interpreting our data. Firstly, significant differences were observed in CR probability during block 3 of the conditioning phase, but these differences did not persist in subsequent blocks. Block 3 occurs early in the conditioning procedure, and participants had a 2–5‐min break before the extinction learning phase, followed by an additional conditioning block where no group differences were detected. Additionally, no other discrepancies in CR properties or UR were observed in this or any other conditioning blocks. Furthermore, the primary results were not influenced by key variables such as age, sex or prior traumatic experiences, indicating effective control of potential confounding factors. These observations suggest that the early differences observed in block 3 were not sustained and were less likely to have impacted the main outcomes. Overall, despite these initial differences, our findings support the conclusion that EM contributes uniquely to accelerating extinction learning within the EBC task.

A second limitation involves the exclusion of data from five participants due to technical issues, including incorrect camera placement on the participant's eye, air pressure problems in the EBC machine and undetected data processing errors. Although this exclusion reduced the sample size, the remaining sample was sufficient to detect significant effects of the EM intervention on extinction learning.

## FUTURE RESEARCH

The findings of this study hold significant potential for advancing future research in several key areas. First, the EBC task offers a valuable tool for uncovering the currently unknown mechanisms underlying EM. It provides a framework to test hypotheses such as the working memory hypothesis, which suggests that EM reduces the vividness of traumatic memories by occupying limited cognitive resources (Engelhard et al., 2011; Wadji et al., 2022). Future studies could further explore how cognitive load during EM interventions influences extinction learning. Second, by repeating the EBC task on multiple days, researchers could investigate the long‐term effects of EM interventions on reconditioning and assess their lasting impact on symptom reduction. This would offer relevant insight into the durability of EM's therapeutic benefits. Third, applying this paradigm to individuals with diverse psychological and psychiatric profiles, such as PTSD patients, could enhance our understanding of individual variability in response to EM interventions. This would help determine the broader clinical applicability of EM across various mental health conditions and settings. Fourth, integrating neuroimaging techniques, such as fMRI or EEG, could provide a deeper understanding of the neural mechanisms at play during EM interventions. By identifying biomarkers of successful extinction learning, researchers could explore the brain regions and pathways involved, such as the cerebellum (Parker et al., 2012; Steiner et al., 2019) and areas linked to working memory (Engelhard et al., 2011; Wadji et al., 2022). In summary, while this study establishes a foundation for understanding the role of EM in extinction learning, future research should employ broader, larger scale, and more targeted methodologies to fully elucidate the therapeutic mechanisms and expand the clinical applications of EM interventions across diverse patient populations and treatment settings.

## CONCLUSION

This study provides evidence supporting the potential efficacy of EM in enhancing extinction learning. The findings affirm that EM has a unique contribution to extinction learning in the EBC task, mirroring observations in EMDR treatment for PTSD. These results offer hope for the use of the EBC task to unravel the yet‐unknown role of EM in the effectiveness of EMDR in treatment settings.

## AUTHOR CONTRIBUTIONS

**Daniel Folch‐Sanchez:** Investigation; writing – original draft; methodology; formal analysis; data curation. **Chrysanthi Blithikioti:** Conceptualization; investigation; funding acquisition; writing – review and editing. **Laura Nuño:** Conceptualization; investigation; methodology; project administration. **Pablo Barrio:** Resources; formal analysis; methodology; writing – review and editing; software. **Roger Borràs:** Data curation; formal analysis; methodology; writing – review and editing; visualization; software. **Laura Blanco:** Conceptualization; investigation; writing – review and editing; project administration. **Flavia Piazza:** Project administration; resources; writing – review and editing. **Mercè Balcells‐Oliveró:** Conceptualization; investigation; funding acquisition; writing – review and editing; project administration; resources; supervision; validation. **Laia Miquel:** Supervision; validation; writing – review and editing; funding acquisition; investigation; conceptualization; project administration; data curation; resources.

## FUNDING INFORMATION

The project ‘HCB/2021/0283’ was funded by Instituto de Salud Carlos III (ISCIII) and co‐funded by the European Union.

## CONFLICT OF INTEREST STATEMENT

The authors declare no conflicts of interest.

## Data Availability

The data supporting the findings of this study will be made available in an appropriate public repository, ensuring open access and guaranteed preservation, upon the acceptance of this manuscript for publication.
